# Hypothesis: Low Vitamin A and D Levels Worsen Clinical Outcomes When Children with Sickle Cell Disease Encounter Parvovirus B19

**DOI:** 10.3390/nu14163415

**Published:** 2022-08-19

**Authors:** Rhiannon R. Penkert, Melissa Azul, Robert E. Sealy, Bart G. Jones, Jola Dowdy, Randall T. Hayden, Li Tang, A. Catharine Ross, Jane S. Hankins, Julia L. Hurwitz

**Affiliations:** 1 Department of Infectious Diseases, St. Jude Children’s Research Hospital, 262 Danny Thomas Place, Memphis, TN 38105, USA; 2 Department of Hematology, St. Jude Children’s Research Hospital, Memphis, TN 38105, USA; 3 Department of Bone Marrow Transplant and Cellular Therapy, St. Jude Children’s Research Hospital, Memphis, TN 38105, USA; 4 Department of Pathology, St. Jude Children’s Research Hospital, Memphis, TN 38105, USA; 5 Department of Biostatistics, St. Jude Children’s Research Hospital, Memphis, TN 38105, USA; 6 Department of Nutritional Sciences, Pennsylvania State University, University Park, PA 16802, USA; 7 Department of Microbiology, Immunology and Biochemistry, University of Tennessee Health Science Center, Memphis, TN 38163, USA

**Keywords:** sickle cell anemia, vitamins A and D, parvovirus B19

## Abstract

Human parvovirus B19 causes life-threatening anemia due to transient red cell aplasia (TRCA) in individuals with sickle cell disease (SCD). Children with SCD experiencing profound anemia during TRCA often require red blood cell transfusions and hospitalization. The prevalence of vitamin deficiencies in SCD is high and deficiencies are associated with respiratory and pain symptoms, but the effects of vitamins on acute infection with parvovirus B19 remain unclear. We performed a clinical study in which 20 SCD patients hospitalized with parvovirus B19 infections (Day 0) were monitored over a 120-day time course to query relationships between vitamins A and D and clinical outcomes. There were significant negative correlations between Day 0 vitamin levels and disease consequences (e.g., red blood cell transfusion requirements, inflammatory cytokines). There were significant positive correlations (i) between Day 0 vitamins and peak virus-specific antibodies in nasal wash, and (ii) between Day 0 virus-specific serum plus nasal wash antibodies and absolute reticulocyte counts. There was a significant negative correlation between Day 0 virus-specific serum antibodies and virus loads. To explain the results, we propose circular and complex mechanisms. Low baseline vitamin levels may weaken virus-specific immune responses to permit virus amplification and reticulocyte loss; consequent damage may further reduce vitamin levels and virus-specific immunity. While the complex benefits of vitamins are not fully understood, we propose that maintenance of replete vitamin A and D levels in children with SCD will serve as prophylaxis against parvovirus B19-induced TRCA complications.

## 1. Introduction

Human parvovirus B19 is a common childhood infection that causes mild, self-limiting symptoms in healthy individuals [1]. However, in individuals with sickle cell disease (SCD), a genetic hemolytic condition, parvovirus B19 can cause life-threatening anemia due to transient red cell aplasia (TRCA). This complication often leads to hospitalization and red blood cell transfusions [2,3]. Limiting the amount of blood transfusions in SCD management is imperative given the high risk of transfusion reaction and allo-immunization [4]. Strategies are needed to prevent complications from parvovirus B19 and reduce disease severity in patients with SCD.

Diseases associated with vitamin deficiencies are vast in number, including xerophthalmia for vitamin A, bone disease for vitamin D, and immune disorders for both [5,6,7,8,9,10,11,12,13,14,15,16,17]. In the general population, low levels of vitamins A and D are associated with increased incidence and severity of viral respiratory infections and decreased immune responses to common viral pathogens [18,19,20,21,22].

Vitamin deficiencies are prevalent in individuals with and without SCD in the United States and worldwide. In one study in Memphis, Tennessee, we found that approximately 50% of participants were vitamin A deficient (VAD) or insufficient, and the majority of participants were vitamin D deficient (VDD) [19]. In one review of individuals with SCD, the prevalence of VDD was reported to range between 56–96% [23]. Low vitamin A or D levels have been associated with SCD complications including low bone mineral density [21], bone fractures [24], high prevalence of vaso-occlusive crises (VOCs) [25], high prevalence of hospitalizations, and severe anemia [26].

That vitamin levels may correlate negatively with infectious disease in healthy populations is well recognized. However, the associations between vitamins, respiratory viral illness, and clinical outcomes in children with SCD have not been fully interrogated. Here we describe a unique prospective observational study with which we define relationships between vitamins A and D, immune responses, and outcomes in children with SCD hospitalized with parvovirus B19-induced TRCA. 

## 2. Materials and Methods

### 2.1. Participant Selection and Clinical Data Collection

Children were enrolled in a single institution iSCREEN protocol (NCT02261480) [27]. The children enrolled on this protocol met the study’s inclusion criteria as follows: a diagnosis of SCD of any genotype, age > 1 year (to avoid the detection of maternal antibodies against parvovirus B19, potentially confounding data interpretation), and hospital admission upon diagnosis of parvovirus B19-induced TRCA, defined as worsened anemia with insufficient compensatory reticulocytosis in the setting of a febrile illness. The acute parvovirus B19 infection was confirmed by a positive diagnostic virus-specific IgM ELISA or polymerase chain reaction (PCR). Participants were excluded if they received chronic erythrocyte transfusion therapy and/or exhibited current epistaxis (which would preclude adequate nasal wash sample collection). In this pilot, observational study, patients were followed and sampled prospectively for 120 days (Days 0, 7, 30, 120), with Day 0 defined as the day of hospital admission. There were some missing samples and/or values for some patients. Clinical and laboratory data were collected prospectively starting on Day 0, and included medications, blood transfusions, and hematologic and hemolytic biomarkers. Absolute reticulocyte count (ARC), hemoglobin, and lactate dehydrogenase (LDH) were measured in a CLIA-certified laboratory at St. Jude Children’s Research Hospital. ARC and hemoglobin were measured on automated hematology analyzers, either Beckman Coulter DxH800 or LH780. LDH was measured with an LDH ver.2 assay and a Roche Cobas 6000 c501 analyzer. This UV assay measured the LDH-mediated conversion of L-lactate to pyruvate (Coefficient of variation [CV] ≤ 2%, SD ≤ 4 U/L). Parvovirus B19 levels were measured using a quantitative real-time PCR assay performed at QUEST Laboratories. Children with SCD generally recovered from acute B19 parvovirus disease and were released from the hospital within 2 to 3 days. The protocol iSCREEN was approved by the St. Jude Children’s Research Hospital Institutional Review Board (IRB, #Pro00004787) with a written consent from the participants’ legally authorized care-giver.

### 2.2. Cytokine/Chemokine Assays

Thirty-eight cytokines/chemokines were measured with a Multiplex (Millipore MAP Kit cat #HCYTMAG-60K-PX38) using a Luminex 200 Multiplexing Instrument and xPonent software. Data were processed using Milliplex Analyst software. Factors included EGF, eotaxin, FGF-2, FKN, Flt-3L, G-CSF, GM-CSF, GRO, IFNa2, IFNγ, IL-1α, IL-1β, IL-1RA, IL-2, IL-3, IL-4, IL-5, IL-6, IL-7, IL-8, IL-9, IL-10, IL12-p40, IL12-p70, IL-13, IL-15, IL-17a, IP-10, MCP-1, MCP-3, MDC, MIP-1α, MIP-1β, sCD40L, TGFα, TNFα, TNFβ, and VEGF. The multiplex assay is non-diagnostic and was used in this study to evaluate relative values. When factors were measured at the lower or upper limit of detection, they were assigned that value for statistical analyses.

### 2.3. Virus-Specific Enzyme-Linked Immunosorbent Assay (ELISA)

ELISAs were performed to measure anti-parvovirus antibody titers in nasal washes (NW) or sera as previously described [27]. Briefly, ELISA plates were coated with 0.1 μg/mL VLP antigen (VP1 and VP2, Prospec cat# prv-002-c; 100 μL/well), washed and blocked. Nasal washes (NW) were diluted 1:10 followed by serial 1:10 dilutions prior to incubations on plates (when antibodies were undetectable at the 1:10 dilution, a titer of 5 was assigned). Sera were diluted 1:100 followed by 1:10 serial dilutions (when antibodies were undetectable at the 1:100 dilution, a titer of 100 was assigned). Washed plates were developed using HRP-conjugated goat anti-human IgG heavy chain (HRP, SBA#2040-05), followed by washes and addition of TMB substrate (KPL). Reactions were stopped with 1 M phosphoric acid. The average OD_450_ readings from samples on uncoated plates were subtracted from test readings and a nonlinear regression program was used to determine antibody titers (GraphPad Prism Version 7, San Diego, CA, USA). A separate diagnostic test was performed by QUEST Diagnostics (Secaucus, NJ, USA) or ARUP Laboratories (Salt Lake City, UT, USA) using a commercial kit (B19V-specific EIAs for B19V-specific IgM and IgG [Biotrin International, Dublin, Ireland]).

### 2.4. Vitamin Measurements

Serum retinol was determined by an internal standard, reverse-phase HPLC method as described previously [19] with photodiode array detection at 325 nm. In brief, aliquots of sera were first saponified to convert all vitamin A to unesterified retinol which was then extracted into hexane. Extraction was performed under conditions of UV-blocked lighting. A precise aliquot of the internal standard, trimethylmethoxyphenyl-retinol, was added and the extract was evaporated to dryness under nitrogen and reconstituted in a small volume (100 µL) of methanol. An aliquot was injected onto a C-18 column and eluted isocratically with a mobile phase of 95% methanol-5% water. The limit of detection was below 0.2 µM plasma and the CV was <5%.

Vitamin D (25 [OH]D) was measured using a Roche Elecsys Vitamin D assay as described previously [19]. This Vitamin D total II assay, an electrochemiluminescence assay, measured D2 and D3 using a Roche Cobas 6000 e601 analyzer (CV ≤ 5.5, SD ≤ 1.1). Retinol binding protein (RBP), often used as a surrogate for vitamin A [28], was measured with a human RBP4 Quantikine kit (R&D Systems, Minneapolis, MN, USA).

We selected cut-offs for vitamin deficiencies and insufficiencies based on Institute of Medicine standards and practice guidelines of the US Endocrine Society. We recognize that these cut-offs remain a topic of debate [29]. We used the values of ≤0.07 μM and >0.07 to ≤1.05 μM retinol to categorize patients as deficient or marginally deficient (insufficient) for vitamin A, respectively. Values of >1.05 μM retinol were considered adequate/replete. The retinol molecular weight of 286.44 was used for unit conversion [5]. For vitamin D, we used <20 ng/mL and ≥20 ng/mL to <30 ng/mL 25(OH)D, respectively, to define deficient and insufficient levels. Values in healthy children can show lower ranges than those of adults [30], although we did not observe a significant correlation between vitamin A or vitamin D levels with age in this small study.

### 2.5. Statistical Analyses

Correlations were evaluated using Spearman’s rank-based correlation (Graphpad Prism Software Version 9). For the comparison of vitamin levels between Days 0 and 120, paired t-tests were used (Graphpad Prism Software Version 9). *p* values were not adjusted for multiple comparisons in this small, observational study.

## 3. Results

### 3.1. Patient Characteristics

Patients hospitalized due to acute parvovirus B19 infection and found to have TRCA were sequentially enrolled over a period of 18 months. Twenty pediatric patients (median age 7 years, range 4.5 to 14.3 years) of different sickle genotypes (15 HbSS, 3 HbSC, 1 HbSD, 1 HbSβeta + thalassemia) were enrolled (Table 1). All patients were African American. The length of hospitalization was no longer than 3 days. All but four patients received packed red blood cell (PRBC) transfusions between Days 0 and 7 inclusive, after which no additional transfusions were required. Among patients who received transfusions, the mean volume by weight was approximately 14 mL/kg.

### 3.2. Laboratory Kinetics during Acute Parvovirus B19 Infection

**Figure 1:**
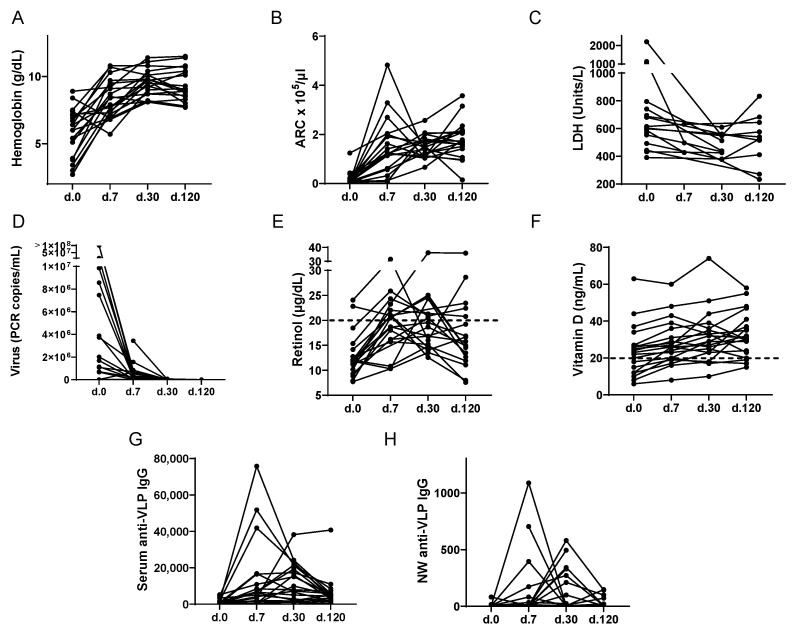
Indices of TRCA Severity and Vitamin Levels Over Time. The X-axis indicates time relative to the day of hospitalization (Day 0, d.0). (**A**–**C**) Hematological trends of 20 pediatric patients with SCD hospitalized due to acute parvovirus B19 infection and found to have TRCA and followed over 120 days. Over the course of 120 days, hemoglobin and reticulocyte counts steadily increased while LDH decreased. (**D**) Parvovirus B19 levels were measured by PCR over 120 days. Almost all patients had undetectable virus levels by Day 120. (**E**) Most patients were vitamin A deficient (retinol < 20 μg/dL) on Day 0. (**F**) Vitamin D (25[OH]D) levels were measured using a Roche Elecsys Vitamin D assay. A dotted line indicates cut-offs for deficiencies. For both vitamin A and vitamin D, levels improved after the acute illness, though many patients remained vitamin A or vitamin D deficient at Day 120. (**G**–**H**) Virus-specific IgG titers in serum and NW samples were followed using a virus-specific ELISA with VLP-coated plates over 120 days.

Indices and kinetics of TRCA disease symptoms, virus loads, vitamins, and virus-specific antibodies are shown in Figure 1 for the 20 patients. There were some missing values. Patients had initial measurements taken at hospitalization (Day 0) with additional measurements taken on Days 7, 30, and 120.

As shown in Figure 1, disease symptoms were measured by hemoglobin, absolute reticulocyte count (ARC), and lactate dehydrogenase (LDH). Hemoglobin and ARC increased while LDH decreased over time (Figure 1A–C). There was often a spike with a subsequent reduction in ARC during the recovery course, as has been previously described [31].

The kinetics of virus load (measured by PCR) varied among patients. Virus levels were highest on Day 0 in most patients and dropped during the time course. At the completion of the study on Day 120, virus was detected in only 2 of 19 tested patients, and values were near the limit of detection (Figure 1D).

Most children were moderately or severely VAD (the latter defined as retinol ≤20 μg/dL), and/or VDD (defined as 25[OH]D < 20 ng/mL, Figure 1E,F). The lowest mean values were on Day 0, when 89% (17/19) of tested patients were VAD and 35% (7/20) of tested patients were VDD. As expected, vitamin A and RBP levels were well correlated. In Supplementary Figure S1 are shown the RBP:retinol correlations on days 0, 7, 30 and 120 (A–D, respectively) and the RBP time course (E). Vitamin levels were significantly improved on Day 120 compared to Day 0 for both vitamin A (paired *t*-test, *p* = 0.013) and vitamin D (paired t-test, *p* = 0.0003), but many children (13/18 or 72% for vitamin A and 3/20 or 15% for vitamin D) remained vitamin deficient on Day 120.

The kinetics of serum and nasal wash (NW) anti-parvovirus B19 IgG antibodies were examined (Figure 1G,H) as previously described [27]. NW antibodies were tested, because the respiratory tract is the most common route for parvovirus B19 infections. At study entry, serum anti-parvovirus B19 IgM and IgG antibodies were first tested with a diagnostic assay [27]. A VLP-based ELISA was then used throughout the time course to monitor antibody titers in both sera and NW. Antibodies generally peaked and waned on different days among patients after hospitalization, perhaps because virus exposures occurred on different days prior to hospitalization.

### 3.3. Correlation between Vitamin Levels and Disease Symptoms upon Hospitalization

**Figure 2:**
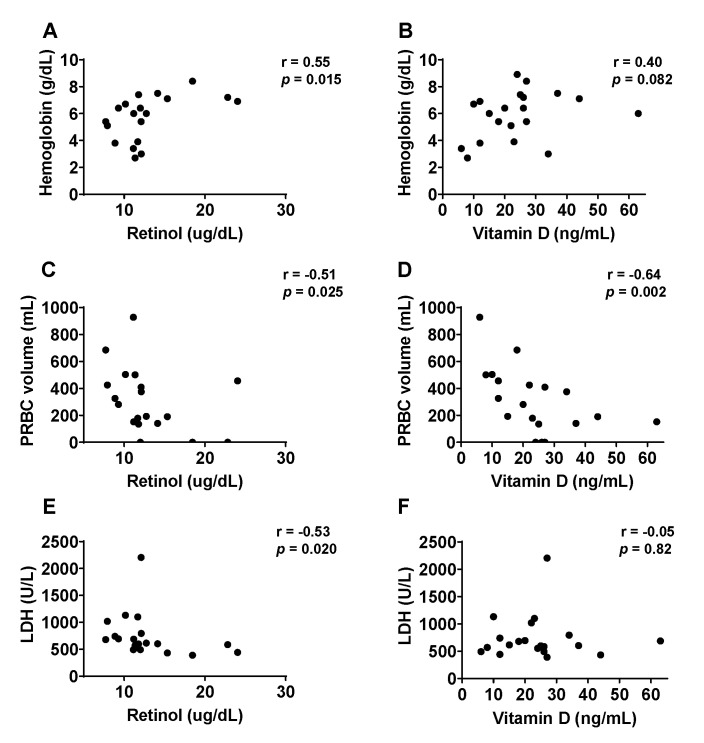
Vitamin levels correlate negatively with TRCA severity. (**A**–**F**) TRCA severity parameters correlations with Day 0 vitamin A (**A**,**C**,**E**) and vitamin D (**B**,**D**,**F**) levels. Associations were analyzed with GraphPad Prism using Spearman’s rank correlation coefficient. We scored significance as *p* < 0.05. Vitamin A was significantly positively correlated with hemoglobin concentration (**A**), and negatively correlated with transfusion volumes (**C**), and LDH (**E**). One retinol measurement was not available. Vitamin D levels significantly, negatively correlated with required PRBC transfusion volumes (**D**). PRBC = packed red blood cells.

We asked if Day 0 disease symptoms could be predicted by Day 0 vitamin levels. As shown in Figure 2, for hemoglobin levels (Figure 2A,B), there was a significant positive correlation with vitamin A (Figure 2A, r = 0.55, *p* = 0.015) and a non-significant positive correlation with vitamin D (Figure 2B, r = 0.40, *p* = 0.082). For PRBC transfusion volumes (Figure 2C,D), there were significant negative correlations, both with vitamin A (Figure 2C, r = −0.51, *p* = 0.025) and vitamin D (Figure 2D, r = −0.64, *p* = 0.002). The lowest hemoglobin level and the two highest PRBC transfusion volumes were in children with both vitamin A and vitamin D deficiencies. For LDH (Figure 2E,F), there was a significant negative correlation with vitamin A (Figure 2E, r = −0.53, *p* = 0.020) and a non-significant negative correlation with vitamin D. It is noteworthy that vitamin D levels that were measured on Day 120, long after PRBC transfusions and patient recovery from TRCA, also correlated negatively with PRBC transfusion volumes (r = −0.52, *p* = 0.020).

### 3.4. Vitamins, Virus, ARC, and the Virus-Specific Antibody Response

We asked if virus-specific antibodies were correlated with Day 0 vitamins, virus and/or the hemolytic marker reflecting symptom severity, ARC. Since virus-specific antibodies were apparently induced at different times among patients, we evaluated antibodies at study entry (Day 0) and also at peak activity (the highest titer among Days 0, 7, 30, and 120 for each patient). As shown in Table 2, significant positive correlations were observed for peak virus-specific IgG in NW with both vitamins A and D on Day 0. Additionally, the retinol value measured on Day 120 had a strong positive correlation with Day 0 diagnostic IgM (r = 0.79, *p* = 0.001), and trended toward a positive correlation with Day 0 diagnostic IgG (r = 0.35, *p* = 0.15). Serum virus-specific IgG (as measured by the diagnostic assay on Day 0) correlated negatively with virus load. Virus-specific IgG from both NW and serum samples (Day 0) correlated positively with ARC.

### 3.5. Cytokine/Chemokine Time Course

**Figure 3:**
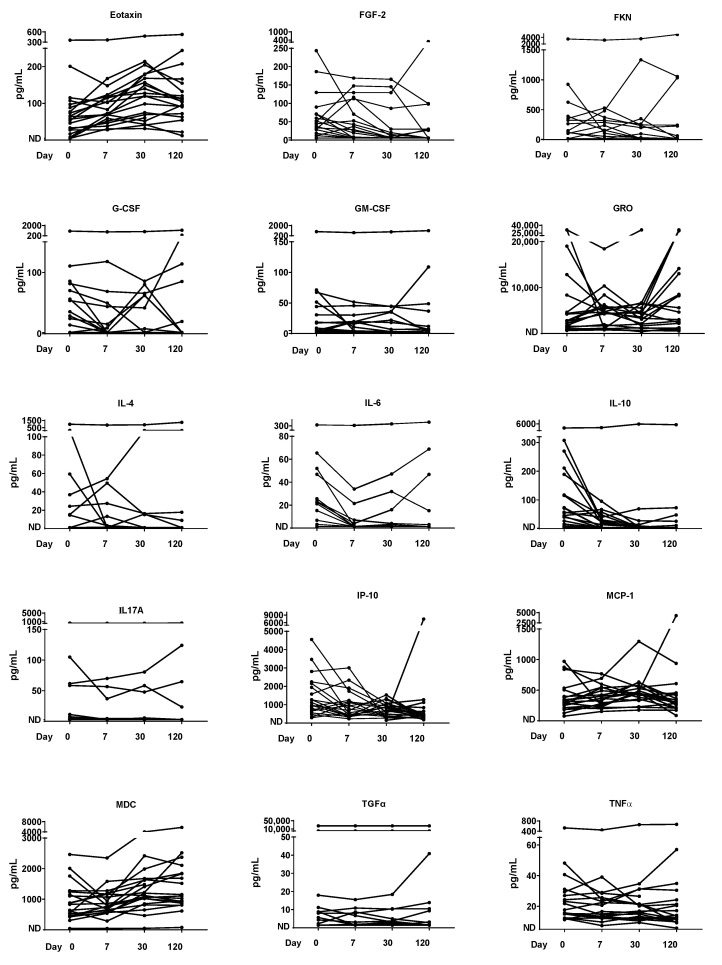
Kinetics of cytokine/chemokine levels. The kinetics of a subset of serum cytokine/chemokine levels are shown. ND = not detected.

We examined cytokines/chemokines over the time-course, as these factors are typically upregulated in patients with SCD [32]. A sampling of kinetics is shown in Figure 3, revealing a variety of patterns. Eotaxin, for example, was increased in most patients between Days 0 and 120, while IL-10 was decreased. One patient showed unusually high values for cytokines/chemokines throughout the study. Complex patterns were expected and noted, given that many different cell types (potentially including virus-specific T cells, dendritic cells, macrophages, fibroblasts, epithelial cells, and developing erythroid cells) secrete cytokines/chemokines during an infection with parvovirus B19 [32,33,34].

### 3.6. Vitamin Correlations with Cytokines/Chemokines

Correlations were tested between vitamins and cytokines/chemokines on Day 0. Table 3 shows all factors for which there were significant correlations with vitamin A or vitamin D. As shown, all significant correlations were negative, and these were more prevalent for vitamin A than for vitamin D. The strongest negative correlations were with IL-17A for both vitamins A and D. The four patients with the highest IL-17A levels (see Figure 3) were all deficient for vitamins A and D.

### 3.7. Virus, ARC and Virus-Specific Antibody Correlations with Cytokines/Chemokines

Correlations between Day 0 virus, ARC, or virus-specific antibodies with Day 0 cytokines/chemokines are shown in Table 4. As demonstrated, IL-10, a factor that can down-regulate immune responses, was positively correlated with virus, and negatively correlated with ARC and virus-specific serum IgG. Additionally, we observed negative correlations for GRO with virus, positive correlations for G-CSF and TGFα with ARC, and negative correlations for IFNγ and IP-10 with virus-specific serum IgG.

### 3.8. Hypothesis

**Figure 4:**
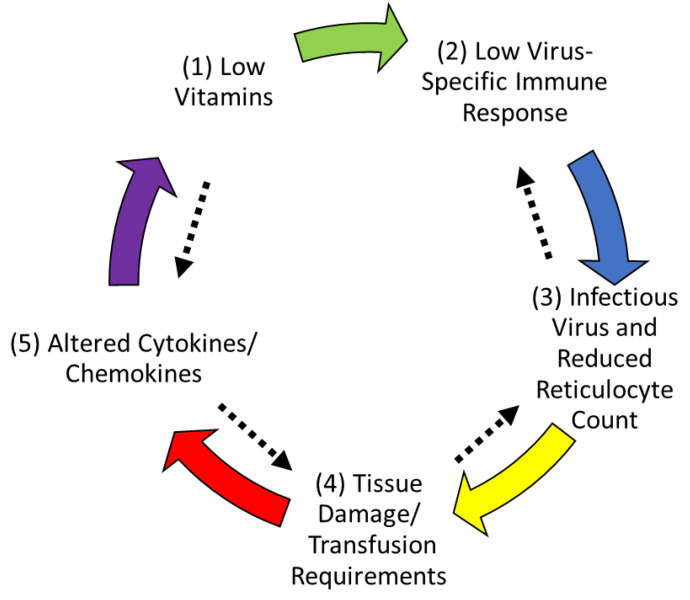
Hypothesis: circular influences among vitamins, the virus-specific immune response, virus amplification, and disease outcome. A hypothesis is illustrated to explain cause-effect relationships as follows: Low vitamin levels (step 1) cause poor virus-specific immune responses (step 2). Poor immunity allows virus amplification and reduced ARC (step 3). Downstream disease consequences, include tissue damage and requirements for transfusions (step 4). Cytokine/chemokine patterns change (step 5) and vitamin levels are further reduced (step 1). Factors are cross-regulatory, and influences are bidirectional (black arrows).

To explain the results, we present a hypothesis that low Vitamin A and D levels are responsible for poor clinical outcomes when children with SCD are exposed to parvovirus B19 (Figure 4). Specifically, we suggest that when a child is suffering hypovitaminosis at the time of a parvovirus B19 exposure (step 1), virus-specific immune responses are poor (step 2). Due to limited immune protection, virus replication progresses, and virus destroys erythroid cell precursors, causing a drop in ARC and a shift in factors associated with erythroid cell mitogenicity/differentiation (step 3) [34,35,36,37]. Downstream disease consequences ensue including higher transfusion requirements (step 4) and changes in cytokine/chemokine profiles (step 5). The process is circular in that altered cytokines/chemokines (step 5) further reduce vitamin levels (step 1). The pathway is bidirectional because factors can cross-regulate (indicated by black arrows). As examples: (i) vitamins may negatively regulate IL-17A-producing cells (Th17) [15,38]) and thereby reduce non-specific inflammation, and (ii) parvovirus B19 may promote IL-10 production to down-regulate virus-specific immunity. In essence, we propose that low baseline vitamin levels set the stage for poor outcomes when children with SCD encounter parvovirus B19.

## 4. Discussion

TRCA is a severe complication in SCD but the modifying effect of vitamin deficiency on clinical outcomes had not been well investigated in previous research. In our study, we investigated the relationship between vitamin levels with severity of TRCA symptoms and inflammatory makers. We found that a large fraction of patients suffered from vitamin deficiencies. There were 89% and 35% patients with deficient levels of vitamins A and D, respectively, upon hospitalization, and despite significant improvements between days 0 and 120, there remained 72% and 15% patients with deficient levels of vitamins A and D, respectively, on Day 120. Low serum vitamin levels (A and/or D) on Day 0 (the day of hospitalization) were associated with greater TRCA severity, including low hemoglobin and a high transfusion requirement in children with SCD and acute parvovirus B19 infection. Vitamins also associated negatively with inflammatory cytokines, particularly IL-17A, consistent with in vitro findings that vitamin A inhibits Th17 cell development [15,38]. There were limitations of the study, including a small sample size. Trends were observed with which hypotheses could be formulated, encouraging a focus on factor relationships and continued confirmatory research.

The hypothesis described in Figure 4 is circular and complex. We propose that low vitamins cause a poor outcome by reducing immune responses to viruses and thereby supporting viral amplification. Viral amplification and consequent disease will further lower vitamin levels, exacerbating the poor outcome [39]. Below, we consider detailed mechanisms and possible prophylaxes/remedies for disease caused by parvovirus B19 in children with SCD.

### 4.1. Low Vitamins Inhibit Virus-Specific Immune Responses and Thereby Increase Virus Amplification and Disease

Studies in research animals have clearly demonstrated that vitamins are required to support healthy virus-specific immune responses and virus control. Vitamins support B cell, T cell, and innate cell activities. Cell development, function, and trafficking are all impaired when vitamin levels are low [6,8,9,10,14,40,41,42,43]. Weakened immune activities can be corrected in research animals by vitamin supplementation [7,11,12,44].

### 4.2. Disease Further Reduces Vitamin Levels

#### 4.2.1. RBP Is an Acute Phase Protein

RBP, an important blood chaperone for vitamin A, is an acute phase protein. Inflammation can induce an acute phase response in which protein synthesis in the liver is either upregulated, as for example for C-reactive protein, or significantly down-regulated, as for a number of nutrient-transport proteins including RBP [45]. In experimental animal studies, administration of lipopolysaccharide (LPS) induced a hepatic response in 12–24 h that included a reduction in mRNA for RBP, and a subsequent reduction in hepatic RBP and plasma holo-RBP levels [46]. This response was transient as, without further administration of inflammatory stimuli, the reduction in RBP synthesis and plasma retinol concentration returned in 3–4 days to normal levels. However, Gieng et al. [47] showed that with a prolonged continuous increase in inflammatory cytokines, the hyporetinolemia persisted. Authors treated rats with a slow-release form of IL-6 and observed a profound persistent reduction in serum holo-RBP. Interestingly, kinetic studies which traced the metabolism of serum retinol provided evidence that the retinol lost from plasma was sequestered out of the plasma compartment, most likely in the liver, and that after the IL-6 treatment ended, the sequestered retinol was available to return to the plasma compartment. Thereafter, the normal rate of plasma retinol turnover was reestablished. These data suggest that inflammation has a profound acute effect on retinol transport, and presumably on its delivery to target tissues, which can become prolonged. The data also suggest that eliminating the inflammation helps to restore plasma retinol concentration and normalize the rate of retinol turnover. Reductions in circulating retinol concentrations have been reported in humans in several inflammatory conditions including infections with measles virus [48], HIV [49], and malaria [50]. Hyporetinolemia is a common feature of many inflammatory conditions and cause-effect relationships may be circular as depicted in Figure 4.

#### 4.2.2. Renal Mechanisms of Hypovitaminosis

Another way in which SCD may affect retinol transport is through impairment of the megalin-mediated reuptake of retinol-RBP from the glomerular filtrate. It is known that the kidneys in SCD are abnormal, and the glomerular filtration rate is high in individuals with SCD (i.e., hyperfiltration) causing increased glomerular capillary pressure and losses in a number of molecules including tubular albumin (albuminuria) [51,52]. Further, recent evidence points toward hemolysis (leading to cell-free hemoglobin) impairing proximal tubule function, a site of vitamin D activation, thereby reducing vitamin D production [53]. Megalin is a large, highly glycosylated extracellularly facing transmembrane protein that is known as a multi-ligand receptor for several nutrient-transport proteins, hormone-binding proteins, and albumin. Its function is to recover these proteins from the glomerular filtrate, which in turn minimizes the loss of micronutrients in the urine. Experimental studies have shown that mice lacking megalin excrete measurable quantities of retinol in urine, whereas this is not observed in wild-type control mice [54,55]. The reuptake of the co-transport protein for RBP, transthyretin, is also impaired [56]. Moreover, megalin together with its cooperating co-protein cubilin also binds vitamin D binding protein (DBP) and is thus a receptor for 25(OH)D. After reuptake of DBP-bound 25(OH)D from the filtrate, DBP is degraded in endocytic vesicles, and the released 25(OH)D molecule can become a substrate for renal CYP27A1, the 1-alpha-hydroxylase in the renal tubular epithelial cells that is responsible for fully activating the vitamin D molecule. Mice lacking megalin become VDD through low renal tubular reuptake and, hence, excessive vitamin D loss in urine [57]. Combining these renal effects of glomerular and tubular damage in SCD, it can be hypothesized that hyperfiltration in the glomerulus of SCD patients, coupled with a lower reuptake in their renal tubular epithelial cells, will significantly enhance losses of vitamin A, vitamin D, and perhaps other micronutrients from the body. Gembello et al. have reported that reduced nephron mass in chronic kidney disease (CKD) is associated with low plasma vitamin D [16] and increased protein loss due to nephrotic syndrome. Moreover, renal impairment or tubular injury is often a cause of chronic inflammation resulting from a combination of oxidative stress, acidosis, and reduced rates of clearance of inflammatory cytokines [58]. It has been hypothesized that hemoglobin negatively affects the reuptake of DBP from the renal filtrate by interfering with the megalin-cubilin-mediated reuptake process [53]. Overall, inflammation disrupts the normal rates of synthesis of several nutrient transport proteins thus interfering with the secretion of micronutrients from liver into plasma, while at the same time inflammation interferes with micronutrient recovery via reuptake in the kidney, such that there may be an overall net loss of micronutrients from the body.

### 4.3. Pediatric Under-Nutrition and Vitamin Supplementation

#### 4.3.1. Under-Nutrition among Children with SCD

Under-nutrition is common in Memphis where this study was performed [20,59,60], and is common in patients with SCD, with increased nutritional requirements [61]. Food insecurity typifies low income neighborhoods where some patients with SCD live [62], meaning these children may have low and insufficient access to micronutrients [63,64,65,66,67]. The consequences of vitamin insufficiencies and deficiencies can be extreme, as vitamins are utilized by virtually every organ system. Vitamins enhance immune function, and low vitamin levels are associated with poor disease outcomes in the context of several respiratory viral infections [7] and asthma [59].

SCD is associated with hypermetabolism and increased resting energy expenditure [66,67,68]. The hypermetabolism in SCD is likely multifactorial and related to chronic hemolysis, increased protein metabolism linked with reticulocytosis, and increased cardiac activity to compensate for severe anemia [64,65,66,67]. Individuals with SCD often have anorexia due to general chronic malaise and frequent hospitalizations [69]. As stated above, many individuals with SCD experience socioeconomic limitations, preventing adequate access to nutrient-rich foods [70,71]. Though ensuring adequate nutrition can be challenging in all children, it may be particularly challenging in those with SCD. Added to challenges are the high frequencies of VDD in African American populations, possibly due to polymorphisms in vitamin D receptors and vitamin D binding proteins [72]. Finally, hyperinflammation induced by acute viral illness, including parvovirus B19, can compound the micronutrient deficiencies of the SCD population as described above. This study underscores the importance of continued efforts to optimize nutrition in patients with SCD.

#### 4.3.2. Benefits/Risks of Vitamin Supplementation

Might vitamin supplementation prevent or treat disease caused by parvovirus B19? A number of vitamin supplementation studies, most often with vitamin D, have been performed and have indicated benefit in patients with SCD, including a reduction in respiratory illness [73,74,75,76,77,78,79,80]. Nonetheless, study results have been mixed. In one small study of patients with SCD, vitamin D supplements were administered orally for six weeks followed by a six-month observation period. Supplements appeared to increase vitamin D levels in sera and decrease days of reported pain by patients, but also decreased health-related quality of life scores [80]. In a randomized controlled clinical trial, children receiving daily vitamin D supplements during winter months had a significantly lower incidence of influenza A compared to placebo controls [81]. In contrast, in a separate study, vitamin D administered at the time of influenza vaccination to healthy children was negatively correlated with immune responses toward the vaccine [19].

In a pilot clinical study of vitamin A supplementation in children with HbSS, improvements in mean corpuscular hemoglobin were noted [77]. However, in research animal studies where an acute phase response was induced by LPS, supplemental vitamin A given orally 6 h after induction of inflammation was not corrective, as most of the vitamin A was retained in the liver [48]. During measles virus infections, vitamin A supplements given to hospitalized children were usually, but not always, protective [17]. In hospitalized children with respiratory syncytial virus (RSV), vitamin A supplements worsened disease [82].

Differing results among studies are influenced by variations in vitamin dose, SCD genotype, patient age, and vitamin status. Conflicting results add to recent articles and meta-analyses that have questioned the overall benefit of vitamin supplements for the prevention of cancer, cardiovascular disease, and death [83,84,85].

Given the variable effects of vitamin supplementation given at the time of disease, we propose that a greater benefit may be gained by establishment of vitamin A plus vitamin D-replete diets (with or without routine vitamin supplements) in children with SCD as prophylaxis against parvovirus B19 disease. The maintenance of adequate vitamin A and D levels as a prophylactic measure may support pathogen-specific immune responses, pathogen control, prevention of disease consequences, and an improved quality of life.

## 5. Conclusions

We describe evidence for poor immune responses and disease outcomes in children with SCD hospitalized with parvovirus B19 TRCA, when baseline vitamin A and D levels are low. We propose that circular relationships are at play. Low vitamin levels at baseline correlate with poor virus-specific immune responses. Poor virus-specific immune responses allow virus to amplify with consequent pathology and changes in cytokine/chemokine profiles. Virus amplification and virus-mediated disease may further reduce vitamin levels and virus-specific immunity. As vitamins are negatively and significantly correlated with SCD pathology post-parvovirus B19 infection, more dedicated vitamin research is needed. Additionally, a concerted effort to establish vitamin replete diets in children with SCD may reduce disease complications when children are exposed to parvovirus B19.

## Figures and Tables

**Table 1:** Characteristics of patients in the iSCREEN study.

Patient	Sex	Age of Patient upon Enrollment (Years)	Sickle Genotype	Retinol (μg/dL)	Vitamin D (ng/mL)
SC001	Male	6.8	SS	7.9	22
SC002	Male	5.5	SS	8.9	12
SC004	Female	7.2	SS	7.7	18
SC005	Male	5.8	SS	12.7	15
SC006	Female	8.5	SC	9.3	20
SC007	Female	6.7	SS	11.7	23
SC008	Male	6.1	SS	11.8	25
SC011	Male	14.3	SS	11.1	6
SC012	Female	9.8	SS	12.0	26
SC013	Male	4.5	SS	14.1	37
SC014	Female	7.2	SS	12.1	34
SC016	Male	10.6	SS	ND	24
SC017	Female	6.2	SS	15.4	44
SC018	Female	11.4	S Beta + Thal	22.8	26
SC019	Male	6.0	SD	11.2	63
SC020	Female	7.0	SS	11.4	8
SC021	Male	11.8	SS	12.1	27
SC022	Female	8.8	SC	10.2	10
SC023	Female	5.3	SS	18.5	27
SC024	Male	12.8	SC	24.1	12

Legend. Patients in the iSCREEN study are described. SS = HbSS, SC = HbSC, S Beta + Thal = HbSβ^+^-thalassemia, SD = HbSD.

**Table 2:** Vitamins, virus load, and absolute reticulocyte count correlations with virus-specific antibodies.

Factor	Day 0 Retinol			Day 0 Vit D			Day 0 Virus			Day 0 ARC		
Virus-specific Antibodies	Corr	*p*	r	Corr	*p*	R	Corr	*p*	R	Corr	*p*	r
Serum Day 0 IgM Diagnostic assay	-----	0.23	0.29	-----	0.58	−0.13	-----	>0.99	−0.14	-----	0.48	0.17
Serum Day 0 IgG Diagnostic assay	-----	0.92	0.03	-----	0.45	0.18	Negative	0.049	−0.49	Positive	0.002	0.65
Serum Peak IgG VLP ELISA	-----	0.17	0.33	-----	0.25	0.27	-----	0.96	−0.01	-----	0.31	0.24
NW Day 0 IgG VLP ELISA	-----	0.38	0.24	-----	0.72	0.10	-----	0.93	0.06	Positive	0.032	0.51
NW Peak Ig GVLP ELISA	Positive	0.020	0.53	Positive	0.047	0.46	-----	0.94	0.02	-----	0.55	0.15

Legend. Correlations (Corr) were with Spearman’s rank-based test. Samples were from sera or NW. Virus-specific antibody values were from Day 0 or from the day of peak titer (Day 0, 7, 30, or 120). Vit D = vitamin D; ARC = absolute reticulocyte count.

**Table 3:** Correlations between vitamins and cytokines/chemokines.

Vitamin	Retinol			Vitamin D		
Factor	Correlation	*p*	r	Correlation	*P*	r
FKN	Negative	0.008	−0.59	-----	0.089	−0.39
Flt-3L	Negative	0.044	−0.47	-----	0.34	−0.22
G-CSF	Negative	0.011	−0.57	-----	0.22	−0.29
GM-CSF	Negative	0.023	−0.52	-----	0.40	−0.20
IFNα2	Negative	0.010	−0.58	-----	0.071	−0.41
IL−1RA	Negative	0.016	−0.55	-----	0.19	−0.31
IL-3	Negative	0.049	−0.46	-----	0.12	−0.36
IL-4	Negative	0.008	−0.59	-----	0.053	−0.44
IL-5	Negative	0.013	−0.56	-----	0.14	−0.34
IL-6	Negative	0.009	−0.58	-----	0.086	−0.39
IL-7	Negative	0.021	−0.53	-----	0.10	−0.38
IL-9	Negative	0.019	−0.53	-----	0.10	−0.38
IL-12p40	Negative	0.003	−0.65	Negative	0.044	−0.45
IL-12p70	Negative	0.006	−0.61	-----	0.071	−0.41
IL-13	Negative	0.025	−0.51	-----	0.22	−0.29
IL-15	Negative	0.003	−0.65	Negative	0.045	−0.45
IL-17A	Negative	0.002	−0.66	Negative	0.005	−0.60
MIP-1b	Negative	0.013	−0.56	-----	0.43	−0.19
TNFα	Negative	0.049	−0.46	-----	0.39	−0.21
TNFβ	Negative	0.015	−0.55	-----	0.12	−0.36

Legend. Correlations were tested between retinol or vitamin D and 38 cytokines/chemokines using the Spearman’s rank-based test. Listed are cytokines/chemokines that yielded a *p* value of <0.05 with either retinol or vitamin D. *p* denotes *p*-value, r denotes rank correlation.

**Table 4:** Virus, ARC, and virus-specific antibody correlations with cytokines.

	Virus			ARC			Serum Virus-Specific IgG (Day 0, Diagnostic Assay)		
Factor	Corr	*p* value	R value	Corr	*p* value	R value	Corr	*p* value	R value
G-CSF	-----	0.41	−0.21	Positive	0.041	0.46	-----	0.74	0.08
GRO	Negative	0.025	−0.56	-----	0.092	0.41	-----	0.12	0.38
IFNγ	-----	0.88	0.04	-----	0.14	−0.34	Negative	0.006	−0.59
IL-10	Positive	0.006	0.64	Negative	0.011	−0.55	Negative	0.003	−0.62
IP-10	-----	0.099	0.41	-----	0.27	−0.26	Negative	0.001	−0.69
TGFα	-----	0.42	−0.22	Positive	0.008	0.59		0.77	0.07

Legend. Correlations (Corr) were tested using Spearman’s rank-based correlation. Factors (Day 0) for which there was a correlation (*p* < 0.05) with virus, ARC, and/or virus-specific antibodies (Day 0; virus-specific IgG antibodies were measured with the diagnostic assay) are shown.

## Data Availability

Data are available upon request to authors.

## Supplementary Materials

The following supporting information can be downloaded at: https://www.mdpi.com/article/10.3390/nu14163415/s1, Figure S1: Retinol correlates positively with retinol binding protein (RBP) A–D. Correlations between retinol and RBP were examined using simple linear regressions throughout the study from Days 0–120. E. The time course for RBP is shown.
