# Supplementary material for: Hypothesis: Low Vitamin A and D Levels Worsen Clinical Outcomes When Children with Sickle Cell Disease Encounter Parvovirus B19
Source: Nutrients. 2022 Aug 19;14(16):3415. doi: 10.3390/nu14163415 (PMC9414848; doi:10.3390/nu14163415)
Supplement: Supplementary file 1 [file nutrients-14-03415-s001.zip › nutrients-1781858-supplementary.pdf]

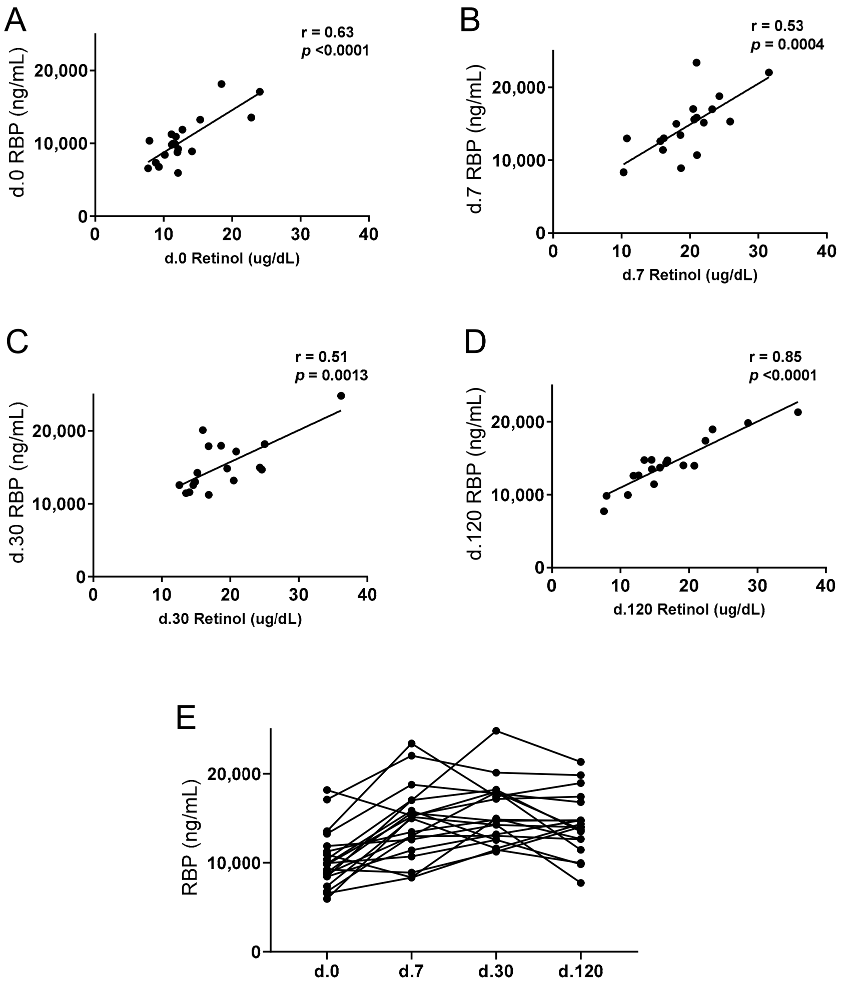

Figure S1. Retinol correlates positively with retinol binding protein (RBP). (**A–D):** Correlations between retinol and RBP were examined using simple linear regressions throughout the study on Days 0, 7, 30, and 120. **(E):** The time course for RBP is shown.
